# Efficacy and toxicity of neoadjuvant chemotherapy versus chemo-immunotherapy in triple-negative breast cancer patients with and without germline BRCA mutations

**DOI:** 10.1007/s10549-026-08006-3

**Published:** 2026-06-22

**Authors:** Tal Etan, Lior Cohen, Yael Bar, Yasmin Leshem, Shir Lerner, Irina Stepansky, Iris Shiran, Amir Sonnenblick, Shlomit Strulov Shachar

**Affiliations:** 1 Oncology Institute, Tel Aviv Medical Center, Tel Aviv, Israel; 2 Gray Faculty of Medical & Health Sciences, Tel Aviv University, Tel Aviv, Israel; 3 Sigma Statistics Ltd., Statistical Consulting Services, Tel Aviv, Israel

**Keywords:** Triple-negative breast cancer, BRCA, Immunotherapy, Neutropenic fever, G-CSF

## Abstract

**Background:**

The addition of pembrolizumab (KN522) to neoadjuvant doxorubicin, cyclophosphamide (AC), carboplatin and paclitaxel (TC) has significantly improved survival, albeit with increased toxicity. This study aims to evaluate the effectiveness and toxicity of KN522 and to decouple the relative contributions of germline BRCA mutations (gBRCAmut) and pembrolizumab addition.

**Methods:**

A retrospective analysis of patients with stage II–III triple negative breast cancer, treated at a single tertiary medical center with either KN522 or ACTC.

**Results:**

Among 127 patients, four cohorts were evaluated: (1) KN522-gBRCAmut (*n* = 26), (2) KN522-BRCA wildtype (*n* = 53), (3) ACTC-gBRCAmut (*n* = 18), (4) ACTC-BRCA wildtype (*n* = 30). The KN522-gBRCAmut cohort achieved a remarkable pCR rate of 92.3%, compared to 60.4%, 55.6% and 36.7% in cohorts (2), (3) and (4) respectively. Multivariable analysis identified the KN522 protocol (OR 3.69, 95% CI 1.66–8.20; *p* = 0.001) and gBRCAmut status (OR 3.78, 95% CI 1.57–9.12; *p* = 0.003) as independent predictors of pCR. Achieving pCR was associated with improved event-free and overall survival (OS) in univariate analyses. In multivariable models, however, pCR remained a significant independent predictor of OS only (HR = 0.15, 95% CI: 0.03–0.72, *p* = 0.018). KN522 was associated with higher hospitalization rates (40.5% vs. 14.6%, *p* = 0.003) and more neutropenic fever (NF) events (32.9% vs. 2.1%, *p* < 0.001), likely due to lower G-CSF prophylaxis during the AC part of the KN522 protocol (21.5% vs. 68.8%, *p* < 0.001).

**Conclusions:**

In this analysis, gBRCAmut carriers treated with KN522 achieved remarkably high pCR rates. The observed 40% hospitalizations, primarily due to NF, highlights the need for supportive care optimization, including G-CSF consideration.

**Supplementary Information:**

The online version contains supplementary material available at 10.1007/s10549-026-08006-3.

## Introduction

Triple-negative breast cancer (TNBC) is an aggressive disease that is curable only when diagnosed at an early stage [1]. The standard of care for stage II and III TNBC involves neoadjuvant systemic therapy with chemo-immunotherapy, followed by surgery, and in some cases, radiotherapy and adjuvant therapy [2]. Despite receiving maximal treatment, approximately one-third of patients experience recurrence, and most of them ultimately die from the disease [1, 3].

The landscape of neoadjuvant therapy for early TNBC has evolved significantly over the past decades. The addition of carboplatin has been shown to increase pathological complete response (pCR) rates [4, 5]. Moreover, the incorporation of pembrolizumab in the KEYNOTE-522 (KN522) protocol has significantly improved distant free survival and overall survival (OS), albeit with increased toxicity [3, 6, 7]. Approximately 80% of patients experienced grade 3 or 4 (G3–4) treatment-related adverse events [6] (AEs), and 82% required protocol modifications due to toxicity [7]. Biomarkers that predict which patients will benefit from the incorporation of immunotherapy into neoadjuvant therapy and which will not, are currently lacking [8].

As both a prognostic marker and a determinant of adjuvant strategy, pathologic complete response (pCR) remains the primary surrogate endpoint for patients with early TNBC undergoing neoadjuvant therapy [9]. Accordingly, tailored adjuvant therapies are recommended for patients with residual disease to further optimize survival [10, 11]. In the KN522 trial, patients who did not achieve pCR demonstrated increased survival benefit from the addition of immunotherapy, compared to the control arm [3].

Approximately 15% of TNBC cases harbor germline BRCA mutations (gBRCAmut), primarily BRCA1 [12]. The prevalence of BRCA carriers among patients with TNBC in Israel is higher, reaching up to 30%, due to the enriched population of Ashkenazi, Sephardic or Ethiopian Jewish descent [13–15]. While gBRCAmut confers high sensitivity to anthracyclines and platinum-based chemotherapy [16–18], its role as a predictor of pCR in the modern neoadjuvant chemo-immunotherapy setting remains inconsistent [19–22]. Specifically, it remains unclear whether the high response rates observed in this population are driven by the addition of immune-checkpoint inhibition or are primarily a manifestation of the inherent chemosensitivity of BRCA-deficient tumors. Furthermore, the impact of immunotherapy addition and BRCA status on the toxicity and tolerability of these intensive regimens has not been adequately characterized in real-world data.

This study aims to compare the efficacy and toxicity of neoadjuvant platinum-based chemotherapy (NAPC) regimens, with or without immunotherapy, to decouple the relative contributions of BRCA status and the addition of pembrolizumab. Such insights are essential for refining clinical decision-making and exploring personalized de-escalation strategies in high-responder subgroups.

## Methods

### Study design and study population

This retrospective cohort study was conducted at Tel Aviv Sourasky Medical Center (TASMC), a tertiary oncology care facility in Israel. Patients with stage II–III TNBC treated at our institution between September 2015 and September 2025 with NAPC were included in the study. Treatment regimens may include either doxorubicin and cyclophosphamide (AC) followed by paclitaxel and carboplatin (TC), or the KN522 protocol (TCAC with pembrolizumab). The administration of the AC part of the protocol in both dose-dense (dd) or every 3 weeks (q3w) regimen was allowed. Patients were excluded if they had an unknown BRCA status, a history of invasive malignancy ≤ 5 years prior to TNBC diagnosis or if they had received prior chemotherapy, targeted therapy, and/or radiation therapy within the 12 months prior to TNBC diagnosis.

### Study end points

pCR (ypT0/Tis, ypN0) was assessed by the local pathologist at the time of definitive surgery and was defined as no residual invasive cancer following completion of neoadjuvant systemic therapy according to the AJCC staging criteria (8th edition). Radiographic response was defined as tumor shrinkage on preoperative sonography or MRI relative to baseline imaging.

Event-free survival (EFS) was defined as the time from first neoadjuvant treatment to any of the following events: progression of disease that precludes surgery, local or distant recurrence, second primary malignancy (breast or other cancers) or death due to any cause.

OS was defined as the time from first neoadjuvant treatment to death due to any cause. Subjects without documented event or death at the time of the analysis were censored at the date of the last follow-up.

Toxicity endpoints included incidence of AEs and immune related AEs (ir-AEs), rates of hospitalizations, and rates of dose interruptions, dose reductions and drug and protocol discontinuations due to AEs. Grading of AEs was determined using the Common Terminology Criteria for Adverse Events (CTCAE) version 5.0 in a scale of Grade (G) 1–5. Relative dose intensity was calculated by the (actual dose delivered per unit time) / (planned dose per unit time) × 100%.

### Data collection

MDclone platform [23] (MDClone, Beer Sheva, Israel) was used to identify all eligible patients. The electronic medical records of these patients were reviewed to collect data on demographics, clinical and pathological tumor features, including age, BRCA status (carrier, wild-type, or unknown), histological subtype (estrogen, progesterone, HER2, and KI67 immunohistochemistry expression level), clinical and pathological stage. Data on neoadjuvant therapy and surgery were also collected, including treatment dates, AC dosing schedule and G-CSF administration, AEs, dose interruptions, dose reductions, drug and protocol discontinuations, and surgery type and timing. Long-term outcomes, including loco-regional recurrence, distant recurrence, second primary malignancy and death, were extracted from the medical records. Unclear cases were revised by an oncologist. The study was approved by the local ethics committee of TASMC institutional review board (TLV-0256-24).

### Statistical analysis

All statistical analyses were performed using IBM SPSS Statistics for Windows, Version 29.0 (IBM Corp., Armonk, NY, USA). A two-sided p-value < 0.05 was considered statistically significant. Descriptive statistics were used to summarize patient demographics, tumor characteristics, treatment details, and clinical outcomes. Categorical variables were reported as frequencies and percentages and compared using the Chi-square test or Fisher’s exact test, as appropriate. Continuous variables were summarized as medians and ranges. Group comparisons of continuous variables were performed using the Mann–Whitney U test.

To evaluate the interplay between genetic status and therapeutic regimen regarding efficacy and safety outcomes, patients were stratified into four cohorts based on treatment protocol and BRCA status: KN522-gBRCAmut (KN522-mut), KN522-BRCA wildtype (KN522-wt), ACTC-gBRCAmut (ACTC-mut), and ACTC-BRCA wildtype (ACTC-wt). These designations are used throughout the manuscript.

Univariate and multivariate logistic regression analyses were conducted to identify independent predictors of pCR, while univariate and multivariate Cox proportional hazards models were used to identify predictors of OS and EFS. All clinical and pathological variables listed in Table 1, alongside relative dose intensity were included in the univariate analyses. For survival prediction, radiological response, surgery type, pCR status, and adjuvant therapy characteristics were also included as covariates. Variables were selected for inclusion in the multivariate models if they demonstrated a p-value < 0.1 in the univariate analysis. Univariate and multivariate logistic regression analyses were conducted to evaluate the association between primary G-CSF prophylaxis and the incidence of febrile neutropenia, as well as febrile neutropenia-related hospitalizations. All statistical tests were two-sided; a p-value < 0.05 was considered statistically significant, and results are reported as Odds Ratios (OR) and Hazard Ratios (HR) with 95% Confidence Intervals (CI).

**Table 1:** Baseline demographic and clinical characteristics by treatment protocol and BRCA status

	KN522 BRCA mut *n* = 26	KN522 BRCA wt *n* = 53	ACTC BRCA mut *n* = 18	ACTC BRCA wt *n* = 30	*p*-value
**Age**					
Median (range)	38.1 (33.6–47.7)	52.4 (42.2–62.5)	37.9 (31.8–45.8)	48.6 (42.1–62.5)	< 0.001
<=50, N (%)	20 (76.9%)	23 (43.4%)	16 (88.9%)	17 (56.7%)	0.001
> 50, N (%)	6 (23.1%)	30 (56.6%)	2 (11.1%)	13 (43.3%)	
**ECOG Performance Status**,** N (%)**					
0	26 (100%)	48 (90.6%)	17 (94.4%)	28 (93.3%)	0.478
1	0 (0%)	5 (9.4%)	1 (5.6%)	2 (6.7%)	
**Clinical T stage**^**a**^, **N (%)**					
T0, Tis, T1	2 (7.7%)	5 (9.4%)	6 (33.3%)	4 (13.3%)	0.379
T2	19 (73.1%)	40 (75.5%)	10 (55.6%)	20 (66.7%)	
T3, T4	5 (19.2%)	6 (11.3%)	2 (11.1%)	4 (13.3%)	
**Clinical N stage**^**a**^, **N (%)**					
N0	7 (26.9%)	20 (37.7%)	7 (38.9%)	11 (36.7%)	0.380
N1	10 (38.5%)	23 (43.4%)	10 (55.6%)	11 (36.7%)	
N2-N3	9 (34.6%)	10 (18.9%)	1 (5.6%)	8 (26.7%)	
**Clinical disease stage**^**a**^, **N (%)**					
Stage II	16 (61.5%)	41 (77.4%)	15 (83.3%)	21 (70.0%)	0.362
Stage III	10 (38.5%)	12 (22.6%)	3 (16.7%)	9 (30.0%)	
**Ki-67**^**b**^, **N (%)**					
<=30%	3 (11.5%)	4 (7.7%)	1 (8.3%)	5 (22.7%)	0.314
> 30%	23 (88.5%)	48 (92.3%)	11 (91.7%)	17 (77.3%)	
**HER2 status**					
0	24 (92.3%)	42 (79.2%)	18 (100%)	24 (80.0%)	0.250
+ 1	2 (7.7%)	5 (9.4%)	0 (0%)	2 (6.7%)	
+ 2/ FISH neg	0 (0%)	6 (11.3%)	0 (0%)	4 (13.3%)	
**AC dosing schedule**					
Every 3 weeks	25 (96.2%)	42 (79.2%)	5 (27.8%)	11 (36.7%)	< 0.001
Dose-dense	0 (0%)	9 (17.0%)	13 (72.2%)	19 (63.3%)	
No AC	1 (3.8%)	2 (3.8%)	0 (0%)	0 (0%)	
**G-CSF**					
Primary prevention	6 (23.1%)	11 (20.8%)	13 (72.2%)	20 (66.7%)	< 0.001
Secondary prevention	6 (23.1%)	18 (34.0%)	2 (11.1%)	4 (13.3%)	
None	14 (53.8%)	24 (45.3%)	3 (16.7%)	6 (20.0%)	
**Surgery type- Breast**,** N (%)**					
Lumpectomy	12 (46.2%)	38 (71.7%)	14 (77.8%)	21 (70.0%)	0.130
Mastectomy	14 (53.8%)	14 (26.4%)	4 (22.2%)	9 (30.0%)	
Axillary surgery alone	0 (0%)	1 (1.9%)	0 (0%)	0 (0%)	
**Surgery type- Axilla**,** N (%)**					
SLNB or TAD	21 (80.8%)	43 (81.1%)	15 (83.3%)	19 (63.3%)	0.230
ALND	4 (15.4%)	10 (18.9%)	2 (11.1%)	9 (30.0%)	
No Axillary surgery	1 (3.8%)	0 (0%)	1 (5.6%)	2 (6.7%)	

^**a**^ Staging according to the American Joint Committee on Cancer (AJCC) 8th edition

^b^ Fifteen patients did not have data regarding their KI67 immunohistochemistry

Abbreviations: AC, doxorubicin and cyclophosphamide; ALND, axillary lymph node dissection; ECOG, The Eastern Cooperative Oncology Group; FISH, Fluorescence in situ hybridization; G-CSF, Granulocyte colony-stimulating factor; HER2, human epidermal growth factor receptor 2; SLNB, sentinel lymph node biopsy; TAD, targeted axillary dissection

pCR rates and their 95% confidence intervals (CIs) were calculated using the Wilson/Brown method. Kaplan–Meier survival analyses were conducted to evaluate OS and EFS using the log-rank test. To address the inherent differences in follow-up duration resulting from the transition in standard-of-care neoadjuvant protocols, survival outcomes were interpreted descriptively. Potential temporal confounding was mitigated by accounting for follow-up time within Cox proportional hazards models, with treatment year included in the regression models to adjust for period-specific effects.

## Results

### Patients and treatment

Overall, 127 patients were eligible for the study and stratified into four cohorts: KN522-mut (*n* = 26), KN522-wt (*n* = 53), ACTC-mut (*n* = 18), and ACTC-wt (*n* = 30). The baseline demographic and pathological characteristics, stratified by treatment protocol and BRCA status are presented in Table 1. Median age was 46 (range 25–82), most patients (83%) had T1-2 tumor (tumor size ≤ 5 cm), and 82 patients (65%) had node positive disease. A total of 44 patients (35%) had gBRCAmut. Median follow-up was 31.5 months (range 6.1–123.6). Most baseline patient and tumor characteristics were similar between four study groups, including ECOG performance status, clinical stage, tumor characteristics and surgery type. (Table 1). Notably, BRCA mutation carriers were significantly younger than BRCA wild-type patients (median age: 38.0 vs. 50.9 years, *p* < 0.001). Significant variations in treatment delivery and supportive care were observed between the two protocols. In the KN522 cohort, the majority of patients (84.8%) were treated with a q3w AC schedule. Conversely, the ACTC group predominantly utilized dose-dense scheduling (66.7%), whereas only 11.4% of the KN522 group received the dose-dense regimen (*p* < 0.001). This shift in scheduling was accompanied by a significantly higher rate of primary G-CSF administration in the ACTC group compared to the KN522 group (68.8% vs. 21.5%, *p* < 0.001). Moreover, since the KN522 regimen was only introduced into clinical practice in recent years, the median follow-up was 62.8 months (IQR 34.43–90.60) for patients treated with ACTC, compared to 23.7 months (IQR 13.47–33.88) for those treated with KN522 (*p* < 0.001).

### Efficacy analysis

**Fig. 1:**
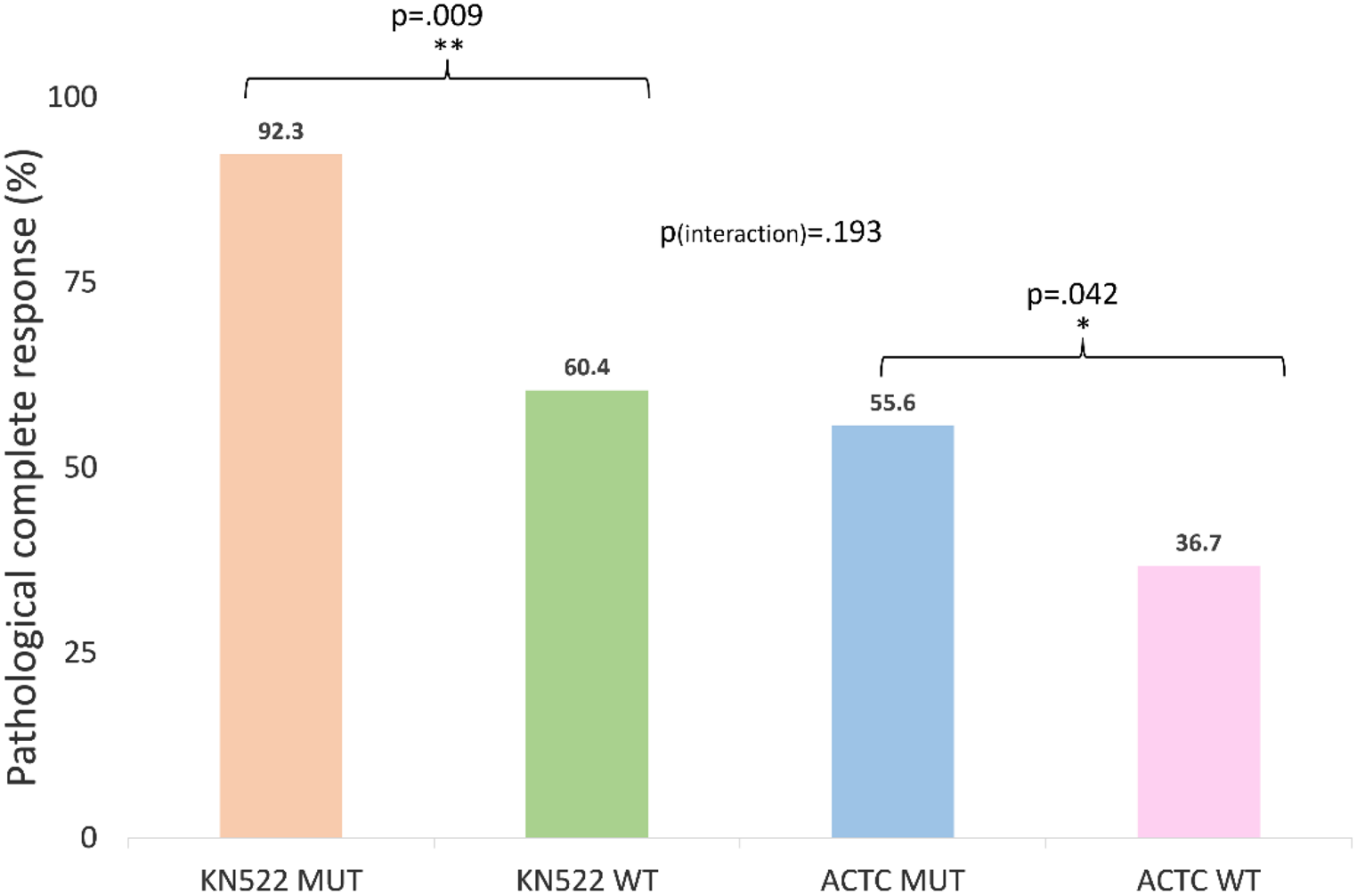
Pathological complete response (pCR) rates stratified by treatment protocol and BRCA status. Bar graph showing the percentage of patients achieving pCR in each subgroup. (*) indicates *p* < 0.05 and (**) indicates *p* < 0.01

Treatment efficacy and clinical outcomes by treatment protocol and BRCA status are presented in Table 2. pCR rates differed significantly across the four study groups (Fig. 1). The KN522-mut cohort achieved a remarkably high pCR rate of 92.3%, compared to 60.4% in the KN522-wt group, 55.6% in the ACTC-mut group, and 36.7% in the ACTC-wt group (*p* < 0.001). While the pathologic response of the primary tumor also varied significantly across these cohorts (*p* = 0.004), the pathologic nodal response did not reach statistical significance (*p* = 0.118). Radiographic response also varied by treatment. While the overall difference in radiographic response rates across the four study cohorts did not reach statistical significance (*p* = 0.135), a significant difference was observed in the radiographic complete response rate between the KN522 and ACTC protocol groups (41.8% vs. 18.8%, *p* = 0.012).

**Table 2:** Treatment efficacy and clinical outcomes by treatment protocol and BRCA status

	KN522 BRCA mut *n* = 26	KN522 BRCA wt *n* = 53	ACTC BRCA mut *n* = 18	ACTC BRCA wt *n* = 30	*p*-value
**pCR, N (%)**					
Yes	24 (92.3%)	32 (60.4%)	10 (55.6%)	11 (36.7%)	< 0.001
No	2 (7.7%)	21 (39.6%)	8 (44.4%)	19 (63.3%)	
**Radiographic response**^**a, b**^, **N (%)**					
Complete Response	12 (50.0%)	21 (39.6%)	4 (25.0%)	5 (17.2%)	0.135
Partial Response	11 (45.8%)	29 (54.7%)	12 (75.0%)	22 (75.9%)	
Stable Disease	1 (4.2%)	3 (5.7%)	0 (0.0%)	1 (3.4%)	
Progressive Disease	0 (0.0%)	0 (0.0%)	0 (0.0%)	1 (3.4%)	
**Pathological T stage**^c^, **N (%)**					
T0 or Tis	24 (92.3%)	32 (60.4%)	11 (61.1%)	15 (50.0%)	0.004
T1 or T1mi	1 (3.8%)	11 (20.8%)	7 (38.9%)	8 (26.7%)	
T2-3	1 (3.8%)	10 (18.9%)	0 (0.0%)	7 (23.3%)	
**Pathological N stage**^c, d^, **N (%)**					
N0	25 (96.2%)	44 (83.0%)	16 (88.9%)	22 (73.3%)	0.118
N1-2	1 (3.8%)	9 (17.0%)	2 (11.1%)	8 (26.7%)	

^**a**^ Radiographic response was defined as tumor shrinkage on preoperative sonography or MRI relative to baseline imaging

^b^ Five patients did not have data regarding radiographic response

^c^ Staging according to the American Joint Committee on Cancer (AJCC) 8th edition

^d^ Patients with N1mic were considered as N1

Abbreviations: pCR; pathological complete response

The univariable and multivariable associations with pCR are summarized in Tables 3 and 4, respectively. In the univariable logistic regression analysis, both gBRCAmut status (OR = 3.16, 95% CI: 1.38–7.23, *p* = 0.006) and the KN522 protocol (HR = 2.73, 95% CI: 1.32–5.63, *p* = 0.007) were significantly associated with an increased likelihood of achieving pCR. These variables remained independent predictors of pCR in the multivariable model. (gBRCAmut: OR 3.78, 95% CI 1.57–9.12; *p* = 0.003 and KN522 protocol: OR 3.69, 95% CI 1.66–8.20; *p* = 0.001).

**Table 3:** Univariate association with pCR

Variable	OR	95% CI	*p*-value
BRCA mutation (Mut vs. WT)	3.16	1.38, 7.23	**0.006**
Treatment (KN522 vs. ACTC)	2.73	1.32, 5.63	**0.007**
Relative dose intensity (%)	1.00	0.98, 1.03	0.827
Time on treatment (weeks)	1.07	0.98, 1.17	0.134
Any G-CSF use (no vs. yes)	1.01	0.49,2.08	0.978
Clinical stage^a^ (II vs. III)	1.19	0.54, 2.63	0.672
T stage^a^ (T0-1 vs. T2-3)	1.36	0.49, 3.81	0.553
N stage^a^ (positive vs. negative)	0.96	0.47, 1.99	0.920
Age (≤ 50 years vs. > 50 years)	1.75	0.86, 3.56	0.120
ECOG performance status (0 vs. 1)	0.38	0.09, 1.66	0.198
KI67 (> 30% vs. ≤ 30%)	1.44	0.45, 4.61	0.536
HER2 expression (+ 1, + 2 vs. 0)	0.71	0.27, 1.88	0.489

^a^ Staging according to the American Joint Committee on Cancer (AJCC) 8th edition

Abbreviations: ALND, axillary lymph node dissection; ECOG, The Eastern Cooperative Oncology Group; G-CSF, Granulocyte colony-stimulating factor; HER2, human epidermal growth factor receptor 2; pCR, pathological complete response; SLNB, sentinel lymph node biopsy; TAD, targeted axillary dissection

**Table 4:** Multivariable association with pCR

Variable	Odds Ratio (OR)	95% CI	*p*-value
BRCA (Mut)	3.78	1.57–9.12	0.003
Protocol (KN522)	3.69	1.66–8.20	0.001

Abbreviations: pCR, pathological complete response

Univariate and multivariable associations with OS and EFS are summarized in Supplementary Tables 1–3. On univariate analysis, achieving pCR and undergoing axillary surgery via SLNB or TAD were significantly associated with improved OS (*p* = 0.006 and *p* = 0.024, respectively) and EFS (*p* = 0.002 and *p* = 0.011, respectively). Radiographic response was associated with EFS only (*p* = 0.028). Notably, neither gBRCA status nor treatment protocol was associated with survival outcomes. In the multivariable model, only pCR remained an independent predictor of OS (HR 0.15, 95% CI: 0.03–0.72, *p* = 0.018), while no significant independent predictors of EFS were observed.

**Fig. 2:**
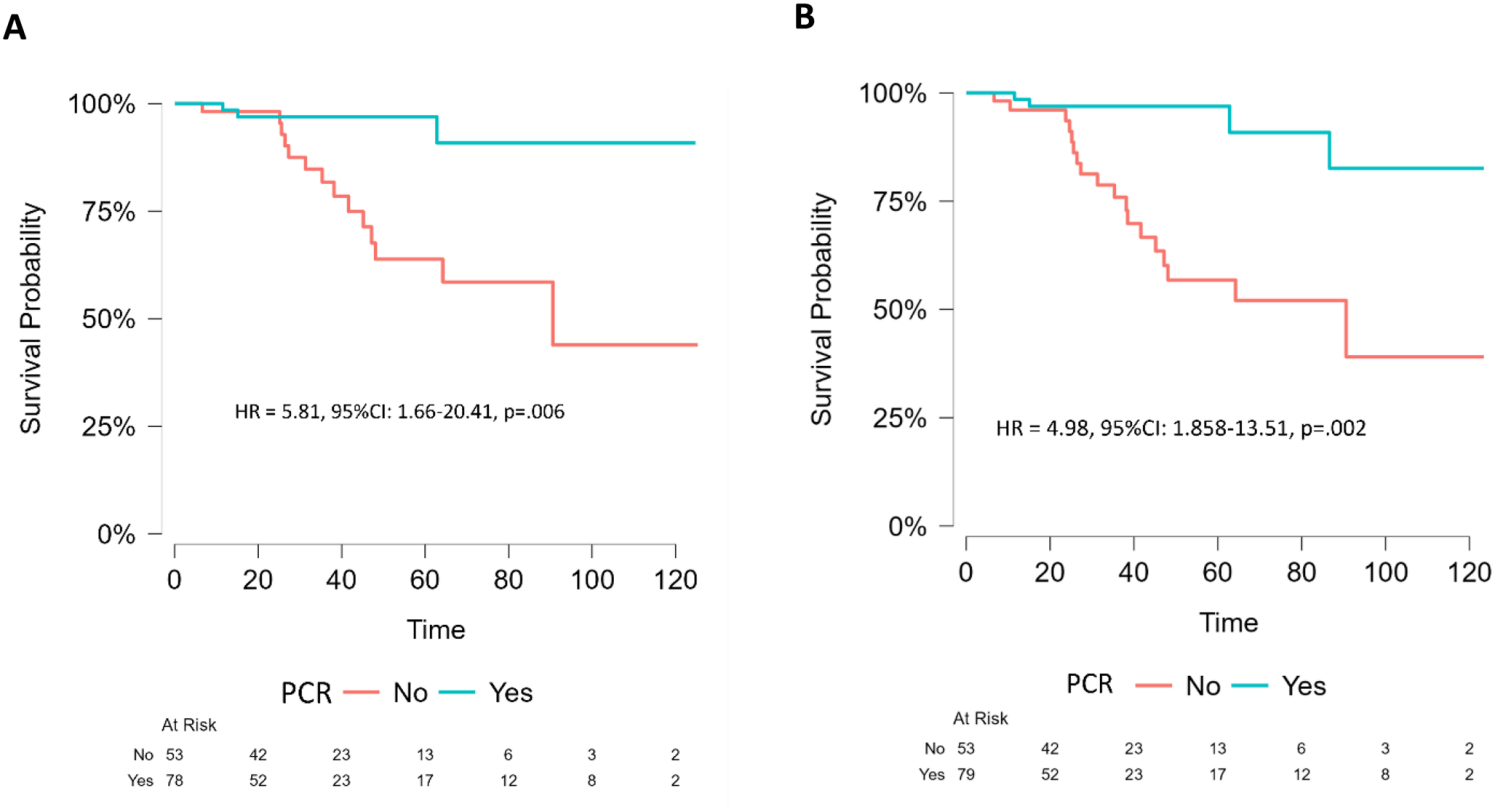
Event-free survival and overall survival curves according to pathological complete response (pCR) status. Panel **A** shows Kaplan–Meier estimates of overall survival according to pCR status. Panel **B** shows Kaplan–Meier estimates of event-free survival according to pCR status

Survival analysis using the Kaplan-Meier method demonstrated that patients achieving pCR had significantly longer OS and EFS compared to those with residual disease (OS: HR = 5.81, 95% CI: 1.66–20.41, *p* = 0.006; EFS: HR = 4.98, 95% CI: 1.858–13.51, *p* = 0.002; Fig. 2A and 2B). In contrast, no significant differences in OS or EFS were observed when comparing the four study cohorts (Supplementary Figs. 1A and 1B).

### Toxicity analysis

The toxicity profiles and neoadjuvant protocol modifications, stratified by treatment arm and gBRCA status, are detailed in Supplementary Tables 4 and 5. All patients (100%) experienced at least one AE, with 63.8% experiencing Grade 3–4 toxicities. The addition of pembrolizumab was associated with 13.9% incidence of G3-4 ir-AEs. The KN522 regimen was associated with a significant increase in hospitalizations and neutropenic fever events (NF) compared to the ACTC group (40.5% vs. 14.6%, *p* = 0.003; and 32.9% vs. 2.1%, *p* < 0.001, respectively). Of the 39 total hospitalizations, 51.3% were attributed to NF and 10.3% to ir-AEs. Other toxicity parameters were comparable across the four study groups including G3-4 AEs (*p* = 0.059), protocol modifications due to AEs (*p* = 0.51) and relative dose intensity (*p* = 0.15). Notably, gBRCAmut status was not associated with an increased risk of G3-4 AEs (*p* = 0.055), ir-AEs (*p* = 0.308) or protocol modifications (*p* = 0.178).

In a univariate logistic regression analyses, primary G-CSF prophylaxis was associated with significantly lower odds of NF-related hospitalizations (OR = 0.29, 95% CI 0.09–0.90, *p* = 0.033) and overall hospitalization (OR = 0.42, 95% CI 0.18–0.96, *p* = 0.039). No significant association was observed between primary G-CSF prophylaxis and the occurrence of NF itself (OR = 0.72, 95% CI 0.30–1.76, *p* = 0.471). In the multivariable logistic regression analyses, primary G-CSF prophylaxis was no longer independently associated with NF, NF-related hospitalization, or overall hospitalization (all p values > 0.05).

## Discussion

This study aimed to compare the efficacy and toxicity of NAPC regimens, with or without immunotherapy, to decouple the relative contributions of BRCA status and the addition of pembrolizumab. Pembrolizumab was associated with significantly higher pCR rates, especially in patients with gBRCAmut, as evidenced by the remarkable 92.3% pCR rate of the KN522-mut group (Fig. 1). Our real-world pCR data are comparable with those reported in the KN522 randomized clinical trial [6], and exceed those observed in other randomized clinical trials [24, 25] and other real-world cohorts [19, 20, 26–28]. Indeed, the achievement of a pCR rate exceeding 90% suggests a potential ‘ceiling effect’ for pathologic response in the gBRCAmut population, raising the question of whether further escalation of neoadjuvant therapy in this subgroup can yield meaningful incremental benefits.

BRCA1 and BRCA2 genes are part of the homologous recombination repair mechanism of double strand DNA breaks and are an important part of maintaining genomic integrity. TNBC tumors with BRCA mutations often exhibit profound genomic instability and are usually more immunogenic than TNBC tumors without these genetic alterations, including increased levels of tumor infiltrating lymphocytes (TILs), higher programmed cell death protein 1 (PD-1) and programmed death-ligand 1 (PD-L1) expression and higher mutational burden [29–31]. High levels of TILs have been shown to be significantly associated with pCR rates with the KN522 regimen [32] and are also associated with better survival [33].

In the pivotal KN522 clinical trial, 78% of patients had positive homologous recombination deficiency (HRD) status, defined as germline or somatic BRCA mutation and/or loss of heterozygosity (LOH) score ≥ 16%. Among them, 17% had gBRCAmut and 4% percent had somatic BRCA mutations. The HRD-positive population achieved higher PCR rates, whether treated with neoadjuvant chemotherapy or chemoimmunotherapy, compared to HRD-negative patients [34]. As somatic BRCA mutations and HRD status are not routinely evaluated in early-stage disease, this study was unable to assess their association with treatment response.

Although both BRCA mutation status and treatment with the KN522 protocol were significantly associated with pCR in univariable and multivariable analyses, and pCR itself was strongly associated with overall survival, neither BRCA status nor treatment protocol appeared to significantly influence survival outcomes. This apparent discrepancy may reflect the complex relationship between pCR and long-term outcomes. One compelling explanation is that, although BRCA mutation carriers tend to have more aggressive tumors [35], they are often diagnosed at earlier stage [36, 37] and achieve higher pCR rates [38, 39] which may attenuate differences in survival. Notably, in the KN522 trial, the addition of immunotherapy improved overall survival primarily among patients who did not achieve pCR [3] suggesting that treatment effects on survival may extend beyond pCR status alone. Our findings are consistent with previous reports and support pCR as a prognostic marker in early TNBC [7, 40, 41]. However, while pCR is generally considered an acceptable surrogate for overall survival, its validity as a surrogate endpoint in BRCA mutation carriers may be more limited [42, 43].

Regarding safety, our real-world cohort demonstrated that the high baseline chemosensitivity and efficacy observed in gBRCAmut carriers did not come at the cost of prohibitive toxicity. Notably, BRCA status was not associated with an increased risk of AEs or protocol modifications. Overall, the toxicity profile observed in our cohort was comparable to that reported in other real-world studies [44, 45]. Importantly 40% of the patients treated with KN522 were hospitalized due to AEs, mostly due to NF. The rate of hospitalizations and NF occurrence was higher than in the pivotal KN522 trial [7]. The elevated incidence of NF may stem from cumulative bone marrow exhaustion caused by the delivery of AC after 12 weeks of TC [5]. This sequence, coupled with the AC dosing schedule and lower rates of primary G-CSF prevention, may underlie the high toxicity observed. While our univariable analysis demonstrated a significant association between lower rates of primary G-CSF prevention and higher NF-related hospitalizations, this significance was lost in the multivariable model. This discrepancy suggests that the protective effect of G-CSF may be masked by broader protocol-related and patient-related factors.

This study is subject to several limitations, most notably its retrospective, single-center design and the inherent differences in treatment eras and follow-up durations between the two protocol cohorts, which may introduce temporal confounding. Furthermore, our survival outcomes (OS and EFS) should be interpreted as descriptive, given the relatively short follow-up period of the KN522 group and the low number of mortality events, these data are currently immature for definitive survival conclusions. Additionally, while our findings regarding pCR are statistically robust, the subgroup analyses, particularly those stratified by gBRCA status, were limited by small sample sizes, which may restrict the statistical power to detect smaller differences in long-term outcomes. Nevertheless, this study provides high-quality real-world insights into treatment efficacy and toxicity. The high proportion of gBRCA carriers in our cohort enhances the relevance of these findings to this distinct population, offering a meaningful contribution to the clinical management of early TNBC.

Altogether, our exploratory findings demonstrate that the KN522 protocol is associated with high pathological response rates, particularly among gBRCAmut carriers. However, the substantial hospitalization and neutropenic fever rates observed in this real-world cohort suggest that the clinical benefit of adding immunotherapy must be balanced against its toxicity profile. Given that the gBRCAmut subgroup achieved excellent outcomes, there is a rationale for investigating neoadjuvant de-escalation strategies in this specific population. Furthermore, the high incidence of febrile neutropenia observed raises the question of whether these outcomes are primarily driven by low G-CSF use or other protocol-related factors, highlighting a critical area for optimization in supportive care for patients receiving the KN522 regimen. While these preliminary results are promising, they warrant further validation in larger, prospectively powered cohorts.

## Supplementary Information

Below is the link to the electronic supplementary material.

Supplementary Material 1

## Data Availability

The data underlying this article are not publicly available due to patient privacy considerations but will be shared by the corresponding author upon reasonable request.
