# Supplementary material for: Efficacy and toxicity of neoadjuvant chemotherapy versus chemo-immunotherapy in triple-negative breast cancer patients with and without germline BRCA mutations
Source: Breast Cancer Res Treat. 2026 Jun 22;217(3):54. doi: 10.1007/s10549-026-08006-3 (PMC13287100; doi:10.1007/s10549-026-08006-3)
Supplement: Supplementary file 1 — Supplementary Material 1 [file 10549_2026_8006_MOESM1_ESM.docx]

**Supplementary Appendix**

Supplementary Table 1. Univariable association with survival

|  |  | **OS** |  |  | **EFS** |  |
| --- | --- | --- | --- | --- | --- | --- |
| **Variable** | **HR** | **95% CI** | **p-value** | **HR** | **95% CI** | **p-value** |
| BRCA mutation (Mut vs WT) | 1.09 | 0.38-3.14 | 0.876 | 1.04 | 0.42-2.56 | 0.932 |
| Treatment (KN522 vs ACTC) | 2.44 | 0.66-9.09 | 0.183 | 1.78 | 0.70-4.48 | 0.224 |
| Treatment year | 0.92 | 0.76-1.12 | 0.402 | 1.09 | 0.93-1.26 | 0.284 |
| Relative dose intensity (%) | 1.00 | 0.96-1.05 | 0.832 | 1.00 | 0.97-1.03 | 0.977 |
| Time on treatment (weeks) | 0.99 | 0.88-1.11 | 0.879 | 1.05 | 0.95-1.16 | 0.360 |
| AC schedule (dose-dense vs q3w) | 1.48 | 0.56-3.90 | 0.431 | 1.08 | 0.46-2.56 | 0.857 |
| Any G-CSF use (yes vs no) | 1.06 | 0.37-3.05 | 0.917 | 1.45 | 0.62-3.37 | 0.390 |
| Clinical stage (III vs II) | 2.27 | 0.86-6.02 | **0.098** | 1.58 | 0.67-3.75 | 0.298 |
| T stage (T2-3 vs T0-1) | 1.73 | 0.40-7.58 | 0.467 | 1.53 | 0.45-5.17 | 0.493 |
| N stage (positive vs negative) | 1.48 | 0.56-3.89 | 0.429 | 1.10 | 0.45-2.68 | 0.830 |
| Age (≤50 vs. >50) | 1.40 | 0.54-3.62 | 0.493 | 1.31 | 0.58-2.99 | 0.516 |
| ECOG performance status (0 vs 1) | 2.13 | 0.48-9.48 | 0.319 | 2.50 | 0.73-8.53 | 0.143 |
| KI67 )>30% vs <30%) | 1.70 | 0.22-13.31 | 0.613 | 2.14 | 0.28-16.30 | 0.462 |
| HER2 expression (+2, +1 vs 0) | 2.75 | 0.89-8.50 | **0.078** | 2.57 | 0.94-6.97 | **0.065** |
| Radiological response (CR vs others) | 5.29 | 0.69-40.00 | 0.108 | 9.62 | 1.28-71.43 | **0.028** |
| pCR (yes vs no) | 5.81 | 1.66-20.41 | **0.006** | 4.98 | 1.85-13.51 | **0.002** |
| Breast surgery (lumpectomy vs mastectomy) | 1.90 | 0.72-5.00 | 0.196 | 2.04 | 0.89-4.67 | **0.094** |
| Axillary surgery (ALND vs. SLNB, TAD) | 3.11 | 1.16-8.31 | **0.024** | 2.99 | 1.29-6.96 | **0.011** |
| Adjuvant Capecitabine | 1.43 | 0.50-4.10 | 0.504 | 1.52 | 0.62-3.71 | 0.36 |
| Adjuvant Pembrolizumab | 0.74 | 0.22-2.45 | 0.618 | 0.52 | 0.20-1.37 | 0.186 |
| Adjuvant Olaparib | 2.71 | 0.81-5.64 | 0.173 | 2.62 | 0.31-22.36 | 0.378 |
| Adjuvant Radiotherapy | 1.35 | 0.18-10.34 | 0.770 | 1.04 | 0.24-4.45 | 0.964 |

^a^ Staging according to the American Joint Committee on Cancer (AJCC) 8th edition

Abbreviations: AC, doxorubicin and cyclophosphamide; ALND, axillary lymph node dissection; CR, complete response; ECOG, The Eastern Cooperative Oncology Group; EFS, event -free survival; FISH, Fluorescence in situ hybridization; G-SCF, Granulocyte colony-stimulating factor; HER2, human epidermal growth factor receptor 2; OS, overall survival; pCR, pathological complete response; SLNB, sentinel lymph node biopsy; TAD, targeted axillary dissection

Supplementary Table 2. Multivariable Cox Proportional Hazards Model for Overall Survival

| **Variable** | **HR** | **95% CI** | **p-value** |
| --- | --- | --- | --- |
| Clinical stage (II vs III) | 0.63 | 0.19-2.04 | 0.442 |
| HER2 expression (0 vs +1-2) | 0.51 | 0.11-2.32 | 0.388 |
| pCR status (pCR vs none-pCR) | 0.15 | 0.03-0.72 | **0.018** |
| Axillary surgery (SLNB or TAD vs ALND) | 0.69 | 0.22-2.08 | 0.518 |

^a^ Staging according to the American Joint Committee on Cancer (AJCC) 8th edition

Abbreviations: ALND, axillary lymph node dissection; HER2, human epidermal growth factor receptor 2; pCR, pathological complete response; SLNB, sentinel lymph node biopsy; TAD, targeted axillary dissection

Supplementary Table 3. Multivariable Cox Proportional Hazards Model for Event-Free Survival

| **Variable** | **HR** | **95% CI** | **p-value** |
| --- | --- | --- | --- |
| HER2 expression (0 vs +1-2) | 0.33 | 0.1-1.12 | 0.076 |
| Radiological response (CR vs non-CR) | 0.16 | 0.02-1.35 | 0.095 |
| pCR status (pCR vs none-pCR) | 0.34 | 0.10-1.11 | 0.076 |
| Breast surgery (mastectomy vs lumpectomy) | 0.57 | 0.22-1.47 | 0.248 |
| Axillary surgery (SLNB or TAD vs ALND) | 0.55 | 0.22-1.36 | 0.204 |

Abbreviations: ALND, axillary lymph node dissection; CR, complete response; HER2, human epidermal growth factor receptor 2; pCR, pathological complete response; SLNB, sentinel lymph node biopsy; TAD, targeted axillary dissection

Supplementary Table 4. Treatment Toxicity Profile by Treatment Protocol and BRCA status

|  | **KN522**  **BRCA MUT**  n=26 | **KN522**  **BRCA WT**  n=53 | **ACTC**  **BRCA mut**  n=18 | **ACTC**  **BRCA wt**  n=30 | **p-value** |
| --- | --- | --- | --- | --- | --- |
| **Ir-AEs, N (%)** | 15 (57.7%) | 38 (71.7%) |  |  | 0.308 |
| **G3-4^a^ AEs, N (%)** | 11 (42.3%) | 39 (73.6%) | 12 (66.7%) | 19 (63.3%) | 0.059 |
| **G3-4 ^a^ ir-AEs, N (%)** | 2 (7.7%) | 9 (17.0%) |  |  | 0.323 |
| **Hospitalization, N (%)** | 7 (26.9%) | 25 (47.2%) | 1 (5.6%) | 6 (20.0%) | **0.003** |
| **Protocol modifications^b^ due to AE, N (%)** | 18 (69.2%) | 43 (81.1%) | 13 (72.2%) | 25 (83.3%) | 0.510 |
| **Protocol modifications^b^ due to ir-AE, N (%)** | 4 (15.4%) | 6 (11.3%) |  |  | 0.722 |
| **Protocol discontinuation due to AE, N (%)** | 2 (7.7%) | 16 (30.2%) | 2 (11.1%) | 5 (16.7%) | 0.071 |
| **One drug discontinuation due to AE, N (%)** | 6 (23.1%) | 14 (26.4%) | 5 (27.8%) | 7 (23.3%) | 0.974 |
| **Dose delay due to AE, N (%)** | 9 (34.6%) | 21 (39.6%) | 4 (22.2%) | 14 (46.7%) | 0.389 |
| **Dose reduction due to AE, N (%)** | 11 (42.3%) | 22 (41.5%) | 4 (22.2%) | 11 (36.7%) | 0.503 |
| **% Relative Dose Intensity of protocol, Median (range)^c^** | 95 (90, 100) | 90 (80, 100) | 97.5 (90, 100) | 90 (90, 100) | 0.151 |

^a^ Grading according to the Common Terminology Criteria for Adverse Events (CTCAE) version 5.0.

^b^ Modifications include dose delay, dose reduction and one or more drug discontinuation.

^c^ Relative Dose Intensity was calculated by the (actual dose delivered per unit time) / (planned dose per unit time) × 100%

Abbreviations: AE, Adverse Event; G, grade; ir-AE, immune-related Adverse Event

Supplementary Table 5. Specific Adverse Events by Treatment Protocol and BRCA status

|  | **KN522**  **BRCA mut**  **N=26** | | **KN522**  **BRCA wt**  **n=53** | | **ACTC**  **BRCA mut**  **n=18** | | **ACTC**  **BRCA WT**  **n=30** | |
| --- | --- | --- | --- | --- | --- | --- | --- | --- |
|  | **Any grade** | **G3-4^a^** | **Any grade** | **G3-4^a^** | **Any grade** | **G3-4^a^** | **Any grade** | **G3-4^a^** |
| **Fatigue, N (%)** | 18 (16.2%) | 0(0%) | 34 (64.1%) | 3 (5.6%) | 9 (50%) | 0(0%) | 21 (70%) | 0(0%) |
| **Anemia, N (%)** | 26 (100%) | 2(7.6%) | 53 (100%) | 13 (24.5%) | 18 (100%) | 3 (16.6%) | 29 (96.6%) | 7 (23.3%) |
| **Neutropenia, N (%)** | 21(80.7%) | 17(65.3%) | 46 (86.7%) | 32 (60.3%) | 16 (88.8%) | 12 (66.6%) | 24 (80%) | 15 (50%) |
| **Thrombocytopenia, N (%)** | 12(46.1%) | 0(0%) | 31 (58.4%) | 7 (13.2%) | 11 (61.1%) | 1 (5.5%) | 19 (63.3%) | 0(0%) |
| **Neuropathy, N (%)** | 12(46.1%) | 3(11.5%) | 32 (60.3%) | 14 (26.4%) | 8 (44.4%) | 1 (5.5%) | 20 (66.6%) | 4 (13.3%) |
| **Nausea, N (%)** | 14 (53.8%) | 1(3.8%) | 24 (45.2%) | 0(0%) | 6 (33.3%) | 0(0%) | 7 (23.3%) | 0(0%) |
| **Vomiting, N (%)** | 1(3.8%) | 0(0%) | 7 (13.2%) | 0(0%) | 1 (5.5%) | 0(0%) | 4 (13.3%) | 0(0%) |
| **Diarrhea, N (%)** | 7(26.9%) | 0(0%) | 16 (30.1%) | 1 (1.8%) | 0(0%) | 0(0%) | 2 (6.6%) | 0(0%) |
| **Skin toxicity, N (%)** | 8(30.7%) | 0(0%) | 16 (30.1%) | 2 (3.7%) | 3 (16.6%) | 0(0%) | 2 (6.6%) | 0(0%) |
| **Hypersensitivity, N (%)** | 2(7.6%) | 0(0%) | 8 (15.09%) | 2 (3.7%) | 4 (22.2%) | 3 (16.6%) | 0(0%) | 0(0%) |
| **Neutropenic fever, N (%)** | 9(34.6%) | 0(0%) | 17 (32.07%) | 0(0%) | 0(0%) | 0(0%) | 1 (3.3%) | 0(0%) |
| **Immune-related Adverse Events** | | | | | | | | |
|  | **KN522**  **BRCA mut**  **N=26** | | **KN522**  **BRCA WT**  **N=53** | | **ACTC**  **BRCA mut**  **n=18** | | **ACTC**  **BRCA WT**  **n=**30 | |
|  | **Any grade** | **G3-4^a^** | **Any grade** | **G3-4^a^** | **Any grade** | **G3-4^a^** | **Any grade** | **G3-4^a^** |
| **Colitis or Gastroduodenitis, N (%)** | 1 (3.8%) | 0 (0%) | 1 (1.8%) | 0 (0%) | --- | --- | --- | --- |
| **Hepatitis, N (%)** | 5 (19.2%) | 1 (3.8%) | 8 (15.09%) | 3 (5.6%) | --- | --- | --- | --- |
| **Pneumonitis, N (%)** | 0 (0%) | 0 (0%) | 2 (3.7%) | 2 (3.7%) | --- | --- | --- | --- |
| **Thyroiditis, N (%)** | 4 (15.3%) | 0 (0%) | 13 (24.5%) | 0(0%) | --- | --- | --- | --- |
| **Dermatitis, N (%)** | 3 (11.5%) | 0 (0%) | 7 (13.2%) | 1 (1.8%) | --- | --- | --- | --- |
| **Carditis, N (%)** | 3 (11.5%) | 0 (0%) | 6 (11.3%) | 0 (0%) | --- | --- | --- | --- |
| **Uveitis, N (%)** | 1 (3.8%) | 0 (0%) | 2 (3.7%) | 1 (1.8%) | --- | --- | --- | --- |
| **Myositis, N (%)** | 0 (0%) | 0 (0%) | 4 (7.5%) | 0 (0%) | --- | --- | --- | --- |
| **Encephalitis, N (%)** | 1 (3.8%) | 1 (3.8%) | 0 (0%) | 0 (0%) | --- | --- | --- | --- |
| **Arthritis, N (%)** | 1 (3.8%) | 0(0%) | 6 (11.3%) | 0 (0%) | --- | --- | --- | --- |
| **Hypophysitis, N (%)** | 1 (3.8%) | 1 (3.8%) | 3 (5.6%) | 2 (3.7%) | --- | --- | --- | --- |

^a^ Grading according to the Common Terminology Criteria for Adverse Events (CTCAE) version 5.0.

Abbreviations: G, grade

**Supplementary Figure 1.**  **Event-Free Survival and Overall Survival Curves According to Treatment Protocol and BRCA Status.**

**A B**

**
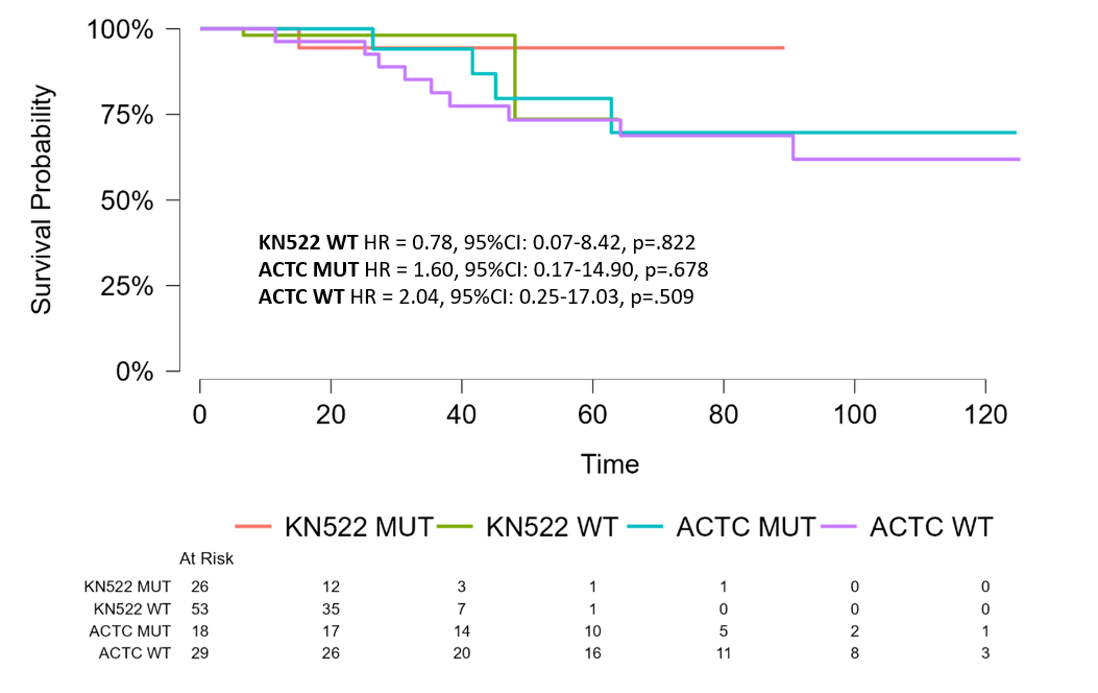

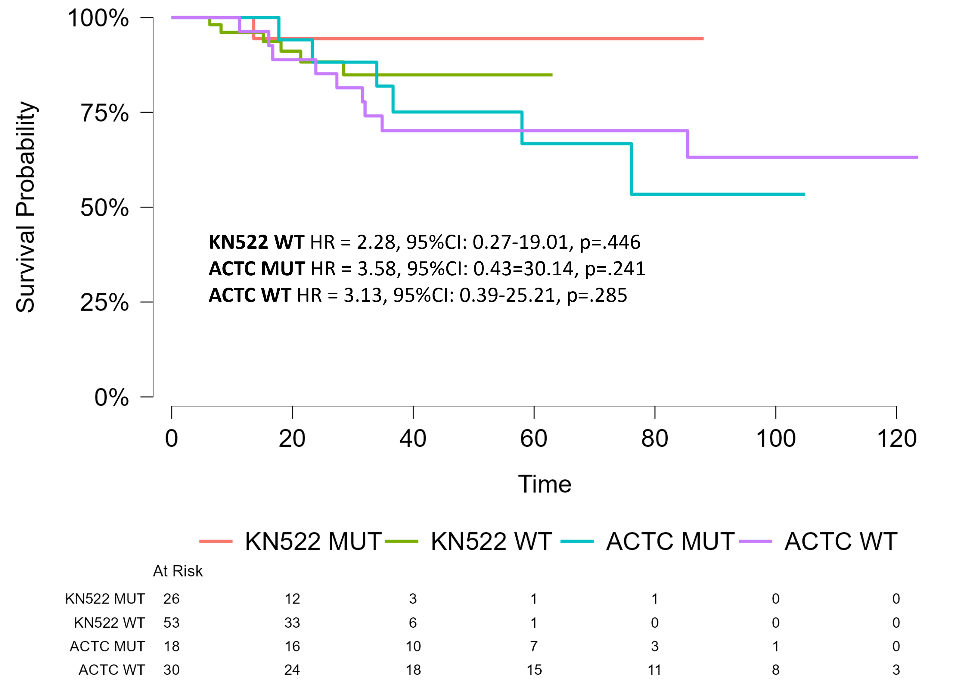
**

**Supplementary Figure 1.**  **Event-Free Survival and Overall Survival Curves According to Treatment Protocol and BRCA Status.** Panel A shows Kaplan–Meier estimates of overall survival according to treatment protocol and BRCA status. Panel B shows Kaplan–Meier estimates of event-free survival according to treatment protocol and BRCA status.
